# Development of a Model to Predict Healing of Chronic Wounds Within 12 Weeks

**DOI:** 10.1089/wound.2019.1091

**Published:** 2020-09-17

**Authors:** Sang Kyu Cho, Soeren Mattke, Hanna Gordon, Mary Sheridan, William Ennis

**Affiliations:** ^1^ Leonard D. Schaeffer Center for Health Policy and Economics, University of Southern California, Los Angeles, California.; ^2^ Center for Economic and Social Research, University of Southern California, Los Angeles, California.; ^3^ Healogics, Inc., Jacksonville, Florida.; ^4^ Department of Surgery, Wound Healing and Tissue Repair Program, University of Illinois Hospital and Health Sciences System, Chicago, Illinois.

**Keywords:** risk scale, prediction, chronic wounds, electronic medical records, real-world data

## Abstract

**Objective:** Chronic wounds represent a highly prevalent but little recognized condition with substantial implications for patients and payers. While better wound care products and treatment modalities are known to improve healing rates, they are inconsistently used in real-world practice. Predicting healing rates of chronic wounds and comparing to actual rates could be used to detect and reward better quality of care. We developed a prediction model for chronic wound healing.

**Approach:** We analyzed electronic medical records (EMRs) for 620,356 chronic wounds of various etiologies in 261,398 patients from 532 wound care clinics in the United States. Patient-level and wound-level parameters influencing wound healing were identified from prior research and clinician input. Logistic regression and classification tree models to predict the probability of wound healing within 12 weeks were developed using a random sample of 70% of the wounds and validated in the remaining data.

**Results:** A total of 365,659 (58.9%) wounds were healed by week 12. The logistic and classification tree models predicted healing with an area under the curve of 0.712 and 0.717, respectively. Wound-level characteristics, such as location, area, depth, and etiology, were more powerful predictors than patient demographics and comorbidities.

**Innovation:** The probability of wound healing can be predicted with reasonable accuracy in real-world data from EMRs.

**Conclusion:** The resulting severity adjustment model can become the basis for applications like quality measure development, research into clinical practice and performance-based payment.

**Figure:**
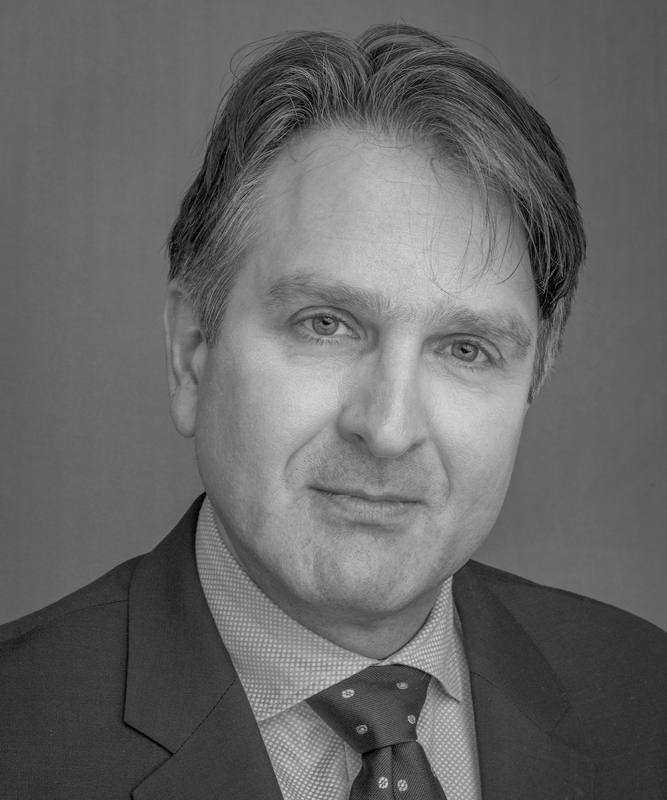
**Soeren Mattke, MD, DSc**

## Introduction

Chronic wounds, usually defined as wounds that do not heal within an expected time frame, typically 4–12 weeks, are a growing but little recognized public health challenge.^1,2^ Their prevalence is similar to that of heart failure, affecting 6.5 million people or 2% of the population in the United States.^3^ In 2014, estimated Medicare expenditure related to managing chronic wounds and associated complications ranged between $28.1 and $96.8 billion.^4^ Patients with chronic wounds often suffer loss of productivity, psychological distress, decreased quality of life, and reduced life expectancy.^5,6^

From advanced dressings to growth factors and biologics, substantial progress has been made in product development and treatment approaches for chronic wounds that do not adequately heal with standard treatment.^7,8^ These advanced wound therapies employ various mechanisms to aid wound healing, such as providing a structural scaffold, repairing restorative cellular mechanisms, increasing delivery of oxygen and nutrients, preventing infection, and removing necrotic tissues or exudates.^9^ However, the uptake of these innovations and use of evidence-based treatment practices remain limited.^1,2,10^

For other chronic conditions, payment reform has been proposed as a policy intervention to improve quality of care because the established fee-for-service payment system is increasingly regarded as failing to reward high-quality care.^4^ The Centers for Medicare and Medicaid Services has charted an ambitious course to reorient its payment models to value and has introduced such models for several chronic conditions.^11^ Several so-called Alternative Payment Models, which link reimbursement to overall resource use, patient outcomes, and adherence to clinical practice guidelines, are tested for conditions like heart failure, diabetes, hypertension, and chronic obstructive pulmonary disease.^12^

## Clinical Problem Addressed

A critical requirement for the introduction of such value-based payment models is scientifically sound and actionable quality indicators that will trigger rewards or penalties. An obvious choice in chronic wound care is the wound healing rate, as closing wounds is both the intended outcome of treatment and readily observable. But given the heterogeneous etiology of chronic wounds, differences in case mix across wound care centers, and the multitude of factors that can influence healing rates proper severity adjustment is needed to construct a valid quality measure.

Several severity adjustment models have been developed previously, but many suffer from limitations such as small sample size from a single center, applicability to only one wound type, such as diabetic foot ulcer, and low prediction accuracy.^13–18^ More recent studies leveraged electronic medical records (EMRs) and machine learning techniques to improve prediction accuracy, but their usability as a quality measure is handicapped by data-driven variable selection and incorporation of information on initial healing observed during follow-up visits.^19,20^

The objective of our study is to develop and validate a unified severity adjustment model for a broad range of chronic wounds that can form the basis for a quality measure as it is entirely based on routinely collected data from intake visits at wound care clinics.

## Materials and Methods

### Dataset

The data for our analyses came from the EMRs of a large network of wound care centers in 46 states and cover the timeframe from January 1, 2014 to September 25, 2018. A detailed description of the staffing and skill mix of these centers has been published elsewhere.^21^ In short, these centers are run by a wound care management company and staffed by combination of employed and contracted physicians, supported by specialized nurses and program managers. All clinicians are required to attend a 1-week specialty wound care training course and are provided evidence based algorithmic clinical practice guidelines. The clinicians subsequently receive ongoing education, monthly conference calls, and have access to regional and local medical directors for case management support. The centers are hospital-based and most have access to specialty consultants and advanced treatment modalities, like hyperbaric oxygen. Standardized treatment protocols promote adherence to evidence-based care.

The data contain detailed patient level information such as age, sex, smoking status, body mass index, comorbid conditions, and wound level measurements such as length, width, and depth in addition to categorical descriptors such as wound etiology, location, and appearance. Each wound is assessed at intake and every subsequent visit, and treatment modalities are documented. At the end of each visit, the outcome for each wound is documented as healed or not healed.

Our starting sample consisted of EMRs for 356,649 patients with 914,878 unique wounds. We excluded 202,842 (22%) wounds that were caused by radiation, acute wounding events such as surgery and trauma, and 71,414 (8%) wounds in patients, who were only seen in the clinic for initial consultation. We removed 20,266 (2%) wounds with implausible dimensions, such as surface areas >100 cm^2^ for arterial ulcer and 150 cm^2^ for other wound types and wounds from patients with missing age and sex.

The final dataset included 620,356 unique wounds from 261,398 patients from 532 wound care clinics. We only used information that was collected during the initial intake for the prediction model and tracked wound outcomes regardless of therapeutic interventions. We selected a training dataset containing a randomly drawn subset of 70% (*n* = 434,249) of the wounds to develop the model, leaving a validation set with the remaining 30% (*n* = 186,107) of the wounds for validation of model parameters (Supplementary Fig. S1).

### Definition of outcome

Our outcome is the status of the wound within the first 12 weeks of treatment initiation, coded dichotomously into healed versus not healed, as commonly used in clinical trials of wound treatment.^22,23^ While patients can remain in treatment for longer than 12 weeks, wound healing in that period was no longer considered for the analysis. Following Ennis *et al.*, we applied a modified intent-to-treat approach in that wounds in patients lost to follow-up during the 12 weeks were considered not healed after excluding wounds that were followed for <7 days.^21^

The determination of healing is made by the treating clinician during each visit based on the following criteria: (1) wound has zero wound measurements, that is, it is completely covered with a full layer of epithelium and no longer has exudate; (2) wound has received a flap procedure and presents post procedure with complete take; (3) wound has received a graft procedure and presents post procedure with complete success; and (4) wound margins have been approximated and sutured to facilitate closure and wound has zero measurements.

### Selection of predictor variables

We combined a review of the published literature on prediction models for wound healing and clinical input to select potential predictor variables. We grouped predictor variables into three categories. The first category represented demographic characteristics, such as age, sex, and smoking status, the second patient level clinical characteristics, such as comorbid conditions and the number of wounds, which were identified based on International Classification of Diseases codes (Supplementary Table S1) or clinical notes, and the third, wound level characteristics, such as area, location, and etiology.

Following Shaw *et al.*, wound area was calculated from measured width and length assuming an elliptical shape.^24^ Physical depth of a wound was estimated by the treating clinicians, and the “anatomical” depth (*i.e.*, degree of tissue penetration) was categorized as partial thickness (including dermis and epidermis but not the fascia), full thickness (extension through the fascia into subcutaneous structure), or unknown thickness based on a classification presented in the Supplementary Data. We refer to this variable as “wound classification.” Variables that were infrequently coded were not considered for the model. For example, prior studies found ankle brachial index, nutritional status, glycated hemoglobin (HbA_1c_), and antibiotic therapy to be predictors of wound healing, but they were infrequently documented in our dataset.^25–27^ While the wound care centers usually collect such information during a patient's intake assessment, it was documented as free text in the EMRs and could not be leveraged for our analyses. ICD-9/10 codes and classification of wound stages are presented in Supplementary Tables S1 and S2.

### Model construction

We used logistic regression models to predict the probability of a wound being healed by the end of week 12 as a function of our three categories of variables: demographic characteristics, patient level clinical characteristics, and wound characteristics. Using the training dataset, we entered variables individually and retained only those that increased the predictive accuracy of the model, as the large sample size implied that many variables were statistically significantly correlated with our outcome without contributing meaningfully to the predictive accuracy.

We added variables in a stepwise fashion and examined their contribution to model performance using area under the curve (AUC) and the Akaike information criterion (AIC). The AUC is a measure of how well the model predicts the outcome.^17^ An AUC of 0.5 means the model performs no better than a random guess, while an AUC of 1.0 means perfect prediction, and values above 0.7 are regarded as acceptable model fit.^28^ Adding variables will always increase predictive power, but also model complexity and risk of “overfitting,” that is, optimizing the model to reflect the information in a specific dataset, while making it less generalizable to different datasets. Hence, the AIC was used to assess the contribution of a variable to the informational quality of the model as it increases, if a variable contributes limited explanatory power relative to its contribution to model fit.^29^

Having selected variables based on examination of AUC and AIC, we constructed three logistic models of ascending complexity: the first model contained only demographic characteristics, the second model added patient-level clinical characteristics, and the final model wound characteristics.

As the magnitude of predicted odds ratios in logistic regression models cannot tell the extent to which each variable drives the prediction, we used a classification tree model with all variables of the final model to further assess the contribution of each explanatory variable.^30^ The classification tree model is a machine learning approach that recursively splits the data into increasingly homogeneous groups using combinations of predictor variables. For example, the algorithm might determine that all wounds below a certain area in nondiabetic women have healed or that pressure ulcers with full thickness in elderly patients that lasted >30 days before admissions did not heal. In other words, the final nodes in the classification tree contain mostly healed or not healed wounds after the data have been split multiple times using combinations of predictor variables. Classification trees can outperform logistic models if there are strong interactions between predictor variables.^31^

The relative variable importance metric derived from such classification tree models is a number between 0 and 1. It is calculated in two steps. First, importance of each variable is measured based on the change in the sum of the squares of residuals (*i.e.*, difference in predicted and actual values) when a predictor variable is used to split a node. Then, relative importance of each variable is calculated by dividing each variable's importance by the highest variable importance among all predictors.^32^ Like logistic models, our classification tree model was developed and validated using 70% and 30% random samples, respectively.

All statistical analyses were conducted using SAS 9.4 (SAS Institute, Cary, NC). Logistic models and classification tree were created using PROC LOGISTIC and PROC HPSPLIT commands, respectively.^33^ The study was reviewed and determined to qualify for the Human Research Protection Program Flexibility Policy by the Institutional Review Board of the University of Southern California (UP-18-00477).

## Results

Table 1 compares characteristics between wounds that healed and wounds that failed to heal by the end of week 12. About 59% of wounds healed by the end of week 12. Because of the large sample size, all variables, except for patient's history of diabetes and chronic obstructive pulmonary disease, showed statistically significant differences, even when the absolute magnitude of the differences was small. Healed wounds were smaller in area and depth and associated with lower prevalence of comorbidities such as Alzheimer's disease or dementia, coronary artery disease, and peripheral vascular diseases. Pressure ulcers and arterial ulcers were less likely to heal than wounds of other etiology.

**Table 1.** Comparison of healed versus not-healed wounds

	Wound Status at 12th Week		
	Healed (*n* = 365,659; 58.9%), n (%)	Not Healed (*n* = 254,697; 41.1%), n (%)	*p*
Demographic characteristics			
Age category			<0.0001
<55	84,840 (23.2)	61,769 (24.3)	
55–64	82,030 (22.4)	57,910 (22.7)	
65–74	89,277 (24.4)	59,054 (23.2)	
>75	109,512 (30.0)	75,964 (29.8)	
BMI category			<0.0001
<18.5	4,180 (1.1)	4,717 (1.9)	
18.5–24	30,725 (8.4)	26,925 (10.6)	
25–29	38,736 (10.6)	28,764 (11.3)	
>30	99,017 (27.1)	54,349 (21.3)	
Missing	193,001 (52.8)	139,942 (54.9)	
Sex (female)	153,052 (41.9)	108,145 (42.5)	<0.0001
Palliative	5,176 (1.4)	10,754 (4.2)	<0.0001
Smoking status			<0.0001
Never smoker	118,640 (32.5)	77,640 (30.5)	
Former smoker	93,523 (25.6)	63,343 (24.9)	
Current smoker	41,226 (11.3)	33,527 (13.2)	
Unknown	112,270 (30.7)	80,187 (31.5)	
Clinical characteristics			
Alzheimer's disease/dementia	13,656 (3.7)	13,920 (5.5)	<0.0001
Coronary artery disease	61,053 (16.7)	46,068 (18.1)	<0.0001
Congestive heart failure	55,559 (15.2)	40,749 (16.0)	<0.0001
Chronic obstructive pulmonary disease	43,703 (12.0)	30,403 (11.9)	0.8586
Diabetes	220,893 (60.4)	154,372 (60.6)	0.1120
Peripheral vascular diseases	77,959 (21.3)	67,471 (26.5)	<0.0001
Quadri/paraplegia	7,681 (2.1)	11,872 (4.7)	<0.0001
Hypertension	189,764 (51.9)	128,880 (50.6)	<0.0001
Number of concurrent wounds			<0.0001
1	169,589 (46.4)	91,799 (36.0)	
2	70,977 (19.4)	52,024 (20.4)	
3 or more	125,093 (34.2)	110,874 (43.5)	
Wound characteristics			
Wound area (cm^2^) (mean/SD)	6.4 (16.5)	9.7 (19.0)	<0.0001
Wound depth (mm) (mean/SD)	2.1 (5.0)	4.1 (8.1)	<0.0001
Wound location			<0.0001
Pelvic	31,801 (8.7)	32,759 (12.9)	
Upper leg	13,345 (3.7)	6,980 (2.7)	
Lower leg	153,995 (42.1)	70,559 (27.7)	
Foot	81,564 (22.3)	87,863 (34.5)	
Toe	53,544 (14.6)	39,462 (15.5)	
Amputation site	3,614 (1.0)	4,199 (1.7)	
Other	27,799 (7.6)	12,875 (5.1)	
Wound classification			<0.0001
Full thickness	157,903 (43.2)	132,153 (51.9)	
Partial thickness	121,364 (33.2)	47,575 (18.7)	
Superficial	30,290 (8.3)	16,710 (6.6)	
Unknown	56,102 (15.3)	58,259 (22.9)	
Wound etiology			<0.0001
Arterial ulcer	8,844 (2.4)	14,842 (5.8)	
Diabetic ulcer	127,129 (34.8)	98,792 (38.8)	
Pressure ulcer	54,500 (14.9)	57,514 (22.6)	
Venous ulcer	97,047 (26.5)	44,035 (17.3)	
Other	78,139 (21.4)	39,514 (15.5)	
Necrotic wound tissue	97,392 (26.6)	88,797 (34.9)	<0.0001
Infected wound	124,198 (34.0)	99,026 (38.9)	<0.0001
Heavily exuding wound	98,921 (27.1)	81,616 (32.0)	<0.0001
Eschar formation	24,500 (6.7)	28,960 (11.4)	<0.0001

BMI, body mass index; SD, standard deviation.

**Figure 1.**
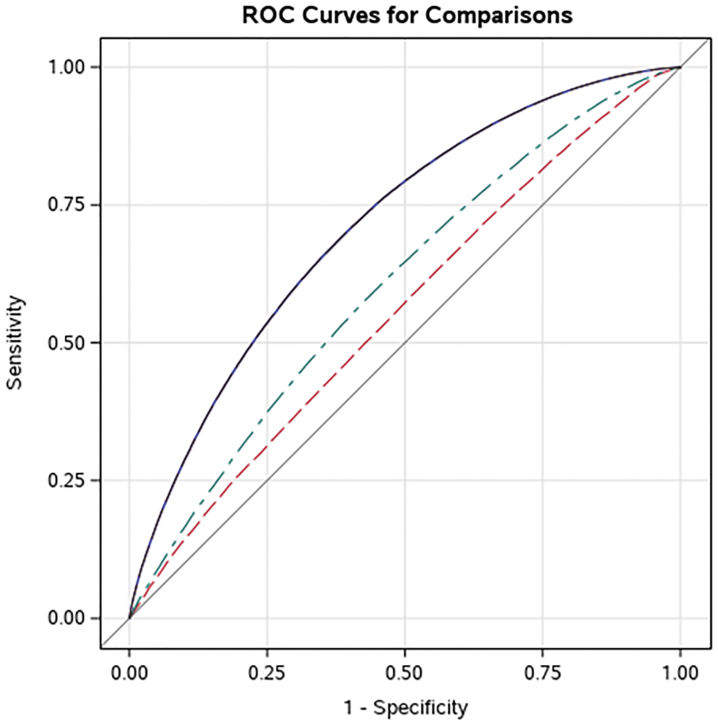
Comparison of area under the receiver operator curves for different model specifications. *Purple*: Model based on demographics + clinical characteristics + wound characteristics. *Green*: Model based on demographics + clinical characteristics. *Red*: Model based on demographics only. ROC, receiver operating characteristic.

The comparison of AUC and AIC for the three logistic regression models is shown in Table 2. The predictive accuracy of a model using only demographic variables was limited with an AUC of 0.556 and adding indicators for comorbid conditions only improved the AUC to 0.605. The addition of variables capturing wound characteristics resulted in substantial improvement in predictive accuracy with an AUC of 0.712. The AIC decreased with the addition of comorbidity and wound characteristic variables, indicating that the additional sets of variables were informative in predicting wound healing. AUCs and AICs of three models are presented in Fig. 1. The comparison of AUCs and AICs between training and validation sets is presented in Supplementary Table S3.

**Table 2.** Comparison of model performance for different specifications

Model Type	AUC	AIC
Model 1: Demographics only	0.556	24,9547.7
Model 2: Demographics + clinical characteristics	0.605	24,5548.4
Model 3: Demographics + clinical + wound characteristics	0.712	22,5519.3

AIC, Akaike information criterion; AUC, area under the curve.

Table 3 shows the estimated odds ratios for the final model that used all wound and patient level characteristics. Pressure ulcers, arterial ulcers, full-thickness wounds, and wounds in patients with multiple concurrent wounds were less likely to heal by the end of week 12.

**Table 3.** Estimated odds ratios from full logistic regression model (Model 3)

Variable (Comparator vs. Reference)	Odds Ratio			
	Point Estimate	95% Confidence Interval		*p*
Depth	0.943	0.942	0.945	<0.0001
Wound surface area	0.987	0.986	0.987	<0.0001
Age category, years				
<55	(Reference)			
55–64	0.999	0.980	1.019	0.0110
65–74	1.028	1.008	1.049	0.0245
>75	1.031	1.011	1.052	0.0064
BMI category				
18.5–24	(Reference)			
25–29	1.091	1.060	1.123	<0.0001
>30	1.277	1.244	1.311	<0.0001
<18.5	0.829	0.782	0.878	<0.0001
Missing	0.968	0.945	0.992	<0.0001
Palliative (yes vs. no)	0.414	0.397	0.433	<0.0001
Sex (male vs. female)	1.125	1.110	1.140	<0.0001
Smoking status				
Never smoker	(Reference)			
Current smoker	0.868	0.849	0.889	<0.0001
Former smoker	0.987	0.969	1.005	<0.0001
Unknown	0.817	0.800	0.834	<0.0001
Dementia/Alzheimer's (yes vs. no)	0.820	0.794	0.848	<0.0001
Coronary artery disease (yes vs. no)	0.981	0.962	1.000	0.0466
Congestive heart failure (yes vs. no)	0.903	0.885	0.921	<0.0001
Chronic obstructive pulmonary disease (yes vs. no)	1.029	1.006	1.051	0.0115
Diabetes (yes vs. no)	1.114	1.095	1.134	<0.0001
Peripheral vascular diseases (yes vs. no)	0.774	0.761	0.787	<0.0001
Quadri/paraplegia (yes vs. no)	0.661	0.634	0.688	<0.0001
Hypertension (yes vs. no)	1.048	1.031	1.066	<0.0001
Number of concurrent wounds				
1	(Reference)			
2	0.728	0.715	0.741	<0.0001
>2	0.567	0.559	0.576	<0.0001
Wound location				
Foot	(Reference)			
Pelvic	1.388	1.349	1.429	<0.0001
Upper leg	2.063	1.982	2.147	<0.0001
Lower leg	1.966	1.928	2.005	<0.0001
Toe	1.319	1.292	1.346	<0.0001
Amputation site	1.117	1.054	1.183	<0.0001
Other	2.019	1.953	2.086	<0.0001
Wound classification				
Superficial	(Reference)			
Full thickness	0.501	0.485	0.517	<0.0001
Partial thickness	0.886	0.856	0.918	<0.0001
Unknown	0.388	0.376	0.402	<0.0001
Wound type				
Diabetic ulcer	(Reference)			
Arterial ulcer	0.669	0.644	0.694	<0.0001
Other	1.609	1.569	1.650	<0.0001
Pressure ulcer	0.857	0.832	0.883	<0.0001
Venous ulcer	1.460	1.425	1.495	<0.0001
Necrotic wound tissue (yes vs. no)	0.784	0.771	0.797	<0.0001
Infected wound (yes vs. no)	0.887	0.874	0.901	<0.0001
Heavily exuding wound (yes vs. no)	0.909	0.894	0.925	<0.0001
Eschar formation (yes vs. no)	0.790	0.770	0.811	<0.0001

The analysis of the relative variable importance based on the classification tree model shows a similar pattern. As shown in Table 4, wound location had the highest variable importance in predicting wound healing, and the importance of the other variables is presented relative to it. Seven of the ten most important predictors were wound level characteristics and eight of the ten least important predictors were patient-level characteristics. Patient level characteristics had a relative variable importance of 0.2 or less, whereas wound characteristics had a relative variable importance around or above 0.5. The classification tree model achieved an AUC of 0.717.

**Table 4.** Relative importance of variables

	Relative Importance
Would location	1.0000
Wound surface area	0.9260
Wound classification	0.7329
Wound etiology	0.5550
Number of concurrent wounds	0.4919
Depth	0.3427
Palliative	0.2441
Necrotic wound tissue	0.2030
Peripheral vascular disease	0.2009
BMI	0.1846
Age	0.1198
Eschar formation	0.1145
Sex	0.1128
Smoking status	0.0943
Infected wound	0.0799
Diabetes	0.0585
Heavily exuding wound	0.0424
Quadri/paraplegia	0.0401
Alzheimer's disease/dementia	0.0343
Congestive heart failure	0.0342
Coronary artery disease	0.0311
Chronic obstructive pulmonary disease	0.0296
Hypertension	0.0262

AUC: 0.717.

## Discussion

Our study used data from a large EMR to predict the probability of wound healing as a function of patient and wound characteristics with both clinically informed and machine learning approaches. Both methods achieved acceptable predictive accuracy with AUCs above 0.70. Our results are consistent with previous findings that wound level characteristics are better predictors of wound healing than patient level characteristics, such as demographic information and comorbidities.^12–14,30^

Our model's performance is comparable or higher than previously published models. For example, using the U.S. Wound Registry that combines EMRs from over 50 wound care clinics, Horn *et al.*^34^ reported AUCs between 0.594 and 0.708 and Fife *et al.*^35^ reported an AUC of 0.648. Using EMRs from a large health system, Margolis *et al.* achieved AUCs of 0.70 and 0.71.^36^ An important distinction, however, is that our model applies to a broad range of wound etiologies, whereas other models were restricted to a single etiology such as diabetic neuropathic foot ulcers or venous leg ulcers.

Another important distinction is that our model exclusively relies on data collected at the patient's initial intake exam, whereas other studies added information on wound healing progress from subsequent visits to increase predictive accuracy. Jung *et al.*, for example, used a machine learning approach and constructed a predictive model with an AUC of 0.842, but the change in wound dimensions between the first and second visit contributed substantially to the model's performance.^19^ Similarly, Horn *et al.* demonstrated that adding information from subsequent visits increased the AUCs to between 0.615 and 0.726 compared to their abovementioned models with AUCs between 0.594 and 0.708 that only used data collected during patient intake.^34^ It is to be expected that the initial healing is a strong predictor of future healing and may help to identify patients, who show insufficient progress in wound healing and may require additional interventions. Thus, models including information on healing progress have utility for clinical decision making. However, the objective of our work was also to use healing rates to compare quality of care between centers. As healing progress will be influenced by initial treatment, we would argue that including it in a model might result in confounding.^37^

Although an AUC >0.70 represents acceptable predictive accuracy of our model, the results point to room for improvement. A logical focus would be information on wound characteristics, as our results show that they are better predictors of healing than patient characteristics. One could explore additional wound level characteristics, such as presence of biofilm, bioburden or activity patterns of metalloproteases, as those have been shown to impact wound healing.^38,39^

In addition, as our results indicate that wound dimensions are an influential predictor of healing, increasing the precision with which they are measured and thus reducing random measurement error might improve predictive accuracy. At the moment, the wound care centers contributing to our data typically estimate surface area based on maximum width and length and approximate depth. Especially for larger and irregularly shaped wounds, this approach can lead to error compared to methods like three-dimensional digital imaging techniques.^40^

Furthermore, more complete documentation of patient-level characteristics could improve predictive accuracy. Body mass index and smoking status, for example, are known predictors of wound healing but were missing in 54% and 31% in our data, respectively.^41^ Similarly, measures of metabolic control, like HbA_1c_, ankle brachial index for perfusion or skin temperature for inflammation are correlated with wound healing but not yet documented routinely in our data.^25,36,42^

Conversely, we have no evidence that increasing the complexity of the model structure will raise predictive accuracy. We tested several interaction effects with only marginal improvement in the AUC. For example, adding interaction terms for wound area × depth and wound location × classification to the final model improved the AUC by only 0.001. Thus, we have no evidence that the interrelationships between predictive variables improve model performance. This result is consistent with the observation that the classification tree model, whose predictive power relies heavily on interactions between predictor variables, had a similar AUC as the final logistic model. Similarly, the use of multi-level models that accounted for clustering of wounds by patients and patients by centers did not improve predictive power meaningfully. In addition, our results suggest that a more parsimonious prediction model using solely wound level characteristics performs comparably to our current model. We tested a logistic model with wound characteristics only, and the model achieved an AUC of 0.689.

Our analysis has important limitations. First, while our dataset is large, it only contains data from one wound care EMR system and would have to be validated, ideally prospectively, with data from other settings. Second, our data have been collected under real-world conditions without validation and/or formal assessment of coding quality and may be subject to measurement error. However, we would argue that this error is likely to be random and thus reduce rather than overstate our predictive accuracy. Lastly, some of our design decisions, such as exclusion of wounds with implausible dimensions and creation of a unified grading scheme, may have introduced error or even bias. As with all predictive models, performance needs to be tracked over time to account for changes in coding practice and clinical care.

## Innovation

Our findings show that wound healing can be predicted based on real-world data from EMRs and a model based on prior evidence and clinical reasoning. Our model can inform clinical decision making and form the basis for applications like quality measure development, future research into clinical practice to determine sources of variability, and performance-based payment.

**Key Findings**

- The probability of healing of chronic wounds within 12 weeks can be predicted with reasonable accuracy using real-world data from EMRs.
- Wound-level characteristics are better predictors of chronic wound healing than patient demographics and comorbidities.
- A machine learning approach using a classification tree model that accounts for complex interaction among predictors variables demonstrates similar accuracy to a logistic regression model.

## Supplementary Material

Supplemental data

## About the Authors

**Sang Kyu Cho, PharmD, MPH,** is a PhD candidate in health economics at Schaeffer Center for Health Policy and Economics at University of Southern California. **Soeren Mattke, MD, MSc,** is a director at Center for Center for Economic and Social Research at University of Southern California. **Hanna Gordon, PhD,** was an employee at Healogics, Inc. at the time this study was conducted. **Mary Sheridan, PhD,** is an employee of Healogics, Inc. **William Ennis, DO, MBA, MMM,** is the section chief of Wound Healing and Tissue Repair Program at University of Illinois.

## Abbreviations and Acronyms

AUC: area under the curve
AIC: Akaike information criterion
BMI: body mass index
EMRs: electronic medical records
HbA_1c_: glycated hemoglobin
ROC: receiver operating characteristic
SD: standard deviation
