# Supplementary material for: Development of a Model to Predict Healing of Chronic Wounds Within 12 Weeks
Source: Adv Wound Care (New Rochelle). 2020 Sep 17;9(9):516–24. doi: 10.1089/wound.2019.1091 (PMC7522633; doi:10.1089/wound.2019.1091)
Supplement: Supplemental data [file Supp_FigS1-TableS1-S3.pdf]

Supplementary Data

Consort diagram

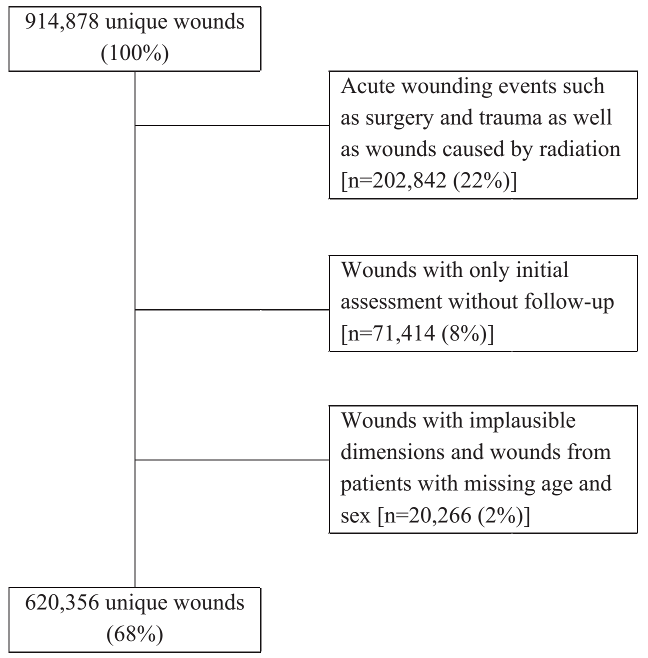

Supplementary Figure S1. Consort diagram.

Supplementary Table S1. *International Classification of Diseases-9/10 codes and clinical codes used for identification of comorbidities*

Comorbidity	*Medical History Item Descriptions in EMR System and ICD-9/10 Codes*
Dementia/Alzheimer's	Dementia<br>331.xx, F03, G30
Coronary artery disease	Coronary artery disease, myocardial infarction<br>410-414.xx, I20-I25
Congestive heart failure	Congestive heart failure<br>428.xx, I50
Chronic obstructive pulmonary disease	COPD<br>490-492.xx, 496.xx, J41-J44
Diabetes	Type I diabetes, type II diabetes, "%Diab%"<br>250.xx, E08-E13, Z79.4
Peripheral vascular diseases	Peripheral arterial disease, peripheral venous disease<br>443.xx, 447.xx, 448.xx, I70, I73, I79.9
Hypertension	Hypertension

COPD, chronic obstructive pulmonary disease; EMR, electronic medical record; ICD, International Classification of Diseases.

Supplementary Table S2. *Classification of wound stages*

EMR Classification	*Model Classification*
NULL	Unknown
Category/stage I	Superficial
Category/stage II	Superficial
Category/stage III	Full thickness
Category/stage IV	Full thickness
Deep tissue pressure injury persistent nonblanchable deep red, maroon, or purple discoloration	Unknown
Eschar covered	Unknown
Full thickness	Full thickness
Full thickness with exposed support structures	Full thickness
Full thickness without exposed support structures	Full thickness
Grade 0	Partial thickness
Grade 1	Partial thickness
Grade 2	Full thickness
Grade 3	Full thickness
Grade 4	Full thickness
Grade 5	Full thickness
Medical device related pressure injury	Unknown
Mucosal membrane pressure injury	Unknown
NA	Unknown
Partial thickness	Partial thickness
Stage 1 Pressure injury	Partial thickness
Stage 2 Pressure injury	Partial thickness
Stage 3 Pressure injury	Full thickness
Stage 4 Pressure injury	Full thickness
Suspected deep tissue injury	Full thickness
Unable to visualize wound bed	Unknown
Unclassifiable	Unknown
Unstageable pressure injury obscured full-thickness skin and tissue loss	Unknown
Unstageable/unclassified	Unknown

NA, not applicable.

Supplementary Table S3. *Area under the curve and Akaike information criterion in training and validation sets*

	Training Set		*Validation Set*	
Model Type	*AUC (95% Confidence Interval)*	*AIC*	*AUC*	*AIC*
Model 1: Demographics only	0.557 (0.555–0.558)	581,902.2	0.556	249,547.7
Model 2: Demographics + clinical characteristics	0.603 (0.601–0.605)	572,943.0	0.605	245,548.4
Model 3: Demographics + clinical + wound characteristics	0.712 (0.710–0.713)	526,368.3	0.712	225,519.3

AIC, Akaike information criterion; AUC, area under the curve.
